# An Analysis of Deterministic Chaos as an Entropy Source for Random Number Generators

**DOI:** 10.3390/e20120957

**Published:** 2018-12-11

**Authors:** Kaya Demir, Salih Ergün

**Affiliations:** TÜBİTAK—Informatics and Information Security Research Center, 41470 Gebze, Kocaeli, Turkey

**Keywords:** deterministic chaos, random number generator, unbounded chaos, bounded chaos, phase-locked loop, Gaussian white noise

## Abstract

This paper presents an analytical study on the use of deterministic chaos as an entropy source for the generation of random numbers. The chaotic signal generated by a phase-locked loop (PLL) device is investigated using numerical simulations. Depending on the system parameters, the chaos originating from the PLL device can be either bounded or unbounded in the phase direction. Bounded and unbounded chaos differs in terms of the flatness of the power spectrum associated with the chaotic signal. Random bits are generated by regular sampling of the signal from bounded and unbounded chaos. A white Gaussian noise source is also sampled regularly to generate random bits. By varying the sampling frequency, and based on the autocorrelation and the approximate entropy analysis of the resulting bit sequences, a comparison is made between bounded chaos, unbounded chaos and Gaussian white noise as an entropy source for random number generators.

## 1. Introduction

Random number generators (RNGs) are fundamental components of cryptographic systems, as they are responsible for generating the unpredictable key values used in ciphering algorithms to protect the integrity, confidentiality and authenticity of the information [1]. Basically, an RNG consists of an entropy source, a sampler to harvest entropy and a post processor to remove statistical imperfections [2]. An ideal entropy source used in an RNG system should have a constant power spectral density over its operating bandwidth, and it is preferable that this bandwidth is as wide as possible [3]. A commonly used entropy source in RNGs is based on amplification of a physical noise in the microscopic domain, such as thermal or shot noise [4,5]. However, it has been previously demonstrated that chaotic noise obtained from a macroscopic system can also be used to generate white noise, eliminating the need for amplification [6]. Despite being deterministic, chaotic systems can be used as an entropy source due to their extreme sensitivity to initial conditions, noise-like power spectrum and positive Lyapunov exponent [7]. The use of chaotic systems as an entropy source in RNGs suggests the possibility of reaching higher throughput data without the need for post processing and with ease of implementation in an integrated circuit form [8,9,10]. With two or more positive Lyapunov exponents, hyperchaotic systems can also be used for random number generation, and they have more complex behaviors, making it harder to predict the RNG output time series [11]. However, synchronization of two coupled hyperchaotic systems despite parameter mismatches was demonstrated in [12] based on the concept of the Master Stability Function. In [13], the security issues of chaos-based random number generators were discussed by studying the synchronization of chaotic systems, and it is suggested that the inclusion of noise analysis in deterministic chaos qualifies chaos-based generators as a truly random number source. In this paper, the use of a phase-locked loop (PLL) device in the chaotic regime for random number generation is considered. The use of PLL circuits for the generation of random numbers in reconfigurable hardware platforms was extensively studied, where the randomness was extracted from the intrinsic jitter of the synthesized clock signal by the PLL [14,15,16,17]. In this paper, the PLL device is used to generate bounded and unbounded chaos, as described in [18,19]. At the input of the voltage-controlled oscillator (VCO) component of the PLL, a chaotic signal is observed under certain conditions; however, the spectrum of this chaotic signal is non-flat over the spectral bandwidth. By adjusting the system parameters, the chaos can be made unbounded, which results in an approximately flat spectrum up to a certain frequency, similar to white Gaussian noise [20]. 

In this paper, using deterministic chaos as an entropy source, random bit sequences of fixed length are generated by regularly sampling the chaotic signal observed at the VCO input when the nature of chaos is bounded and unbounded. The sampling frequency limits the maximum throughput of the RNG and is a critical parameter to ensure randomness in the generated bit stream. A faster sampling rate is preferred for high-throughput data, but the bandwidth of the entropy source imposes restrictions on the maximum allowable sampling frequency to maintain randomness in the resulting bit sequence. To investigate this phenomenon, the sampling frequency is gradually elevated, and the randomness of the resulting bit streams are assessed through the application of the concepts of autocorrelation and approximate entropy. Then the bounded and unbounded chaos is benchmarked against white Gaussian noise, which might originate in a stationary stochastic process. It is numerically shown that up to a certain frequency which is dependent on the PLL system parameters, unbounded chaos approaches white Gaussian noise as an entropy source to generate random numbers by the regular sampling of an irregular waveform method. To the best of authors’ knowledge, this is the first analytical study on the application of bounded and unbounded chaos from a PLL device as an entropy source for RNG and the comparison of deterministic bounded or unbounded chaos and white Gaussian noise from a stochastic process. This paper is organized as follows. In Section 2, the equations governing the PLL system are described. Section 3 focuses on the formation of bounded and unbounded chaos in PLL and the analysis of the associated chaotic signals. In Section 4, regular sampling of the chaotic signal method is applied to generate random bit sequences using bounded chaos, unbounded chaos and white Gaussian noise as an entropy source and the randomness of the generated bit sequences are discussed, followed by conclusions in Section 5.

## 2. Chaotic System

**Figure 1:**
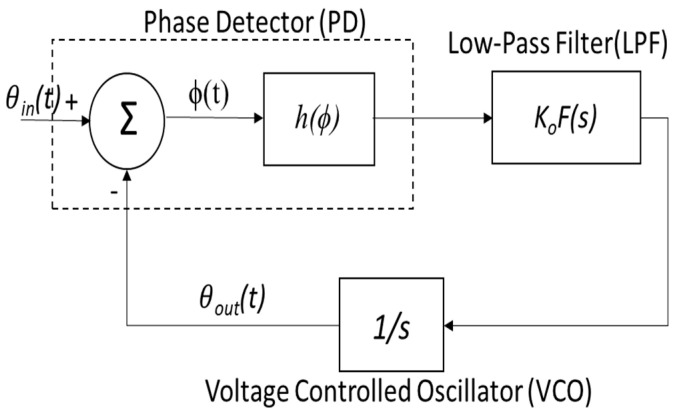
The phase model of the PLL system.

The chaotic system in this paper is based on a sinusoidally driven PLL, which has previously been extensively studied in [19,20]. Therefore, the equations will be summarized by referring to [19,20]. Basically, a PLL device is made up of a phase detector to identify the phase error, a low-pass filter and a VCO generating a square wave at a frequency dependent on input signal amplitude. Figure 1 illustrates the phase model of a PLL. The $\theta_{in},\;\theta_{out}$ and $\phi \left(t\right)=\theta_{in}-\theta_{out}$ are input phase, output phase and phase error, respectively. 

The nonlinear function *h(ϕ)* is a 2π-periodic function of *ϕ* and it is in symmetric triangular form for an EX-OR type phase detector. It is defined as

(1) h(ϕ)=h(ϕ+2nπ), n=0,±1,±2,…..n∈ ℤ

(2) h(ϕ)={ϕ       for |ϕ|<π2−ϕ+π for  π2<ϕ<3π2 

The transfer function of the low-pass (lag-lead) filter is given as

(3) $$F\left(s\right)=\left(1+\tau_{2}s\right)/\left(1+\tau_{1}s\right)$$

Following the diagram in *s*-domain

(4) $$\phi \left(s\right)=\theta_{\mathrm{in}}\left(s\right)-\theta_{\mathrm{out}}\left(s\right)$$

(5) θout(s)=h(ϕ(s))K0F(s)· 1s

Substituting (3), (5) in (4), using cross multiplication and then dividing each side by $\tau_{1}$,

(6) $$\phi \left(s\right)+h\left(\phi \left(s\right)\right)K_{0}\frac{\left(1+\tau_{2}s\right)}{\left(1+\tau_{1}s\right)s}=\theta_{in}\left(s\right)$$

(7) $$\tau_{1}s^{2}\phi \left(s\right)+s\phi \left(s\right)+K_{0}h\left(\phi \left(s\right)\right)+\tau_{2}sK_{0}h\left(\phi \left(s\right)\right)=\tau_{1}s^{2}\theta_{in}\left(s\right)+s\theta_{in}\left(s\right)$$

(8) $$s^{2}\phi \left(s\right)+\frac{1}{\tau_{1}}\left(s\phi \left(s\right)+K_{0}\tau_{2}sh\left(\phi \left(s\right)\right)\right)+\left(\frac{K_{0}}{\tau_{1}}\right)h\left(\phi \left(s\right)\right)=s^{2}\theta_{in}\left(s\right)+\frac{1}{\tau_{1}}s\theta_{in}\left(s\right)$$

Using (8), with respect to the phase error *ϕ*, the system equation in the time domain can be stated as

(9) $$\frac{d^{2}\phi }{dt^{2}}+\frac{1}{\tau_{1}}\left(1+K_{0}\tau_{2}h^{\prime }\left(\phi \right)\right)\frac{d\phi }{dt}+\left(\frac{K_{0}}{\tau_{1}}\right)h\left(\phi \right)=\frac{d^{2}\theta_{in}}{dt^{2}}+\frac{1}{\tau_{1}}\frac{d\theta_{in}}{dt}$$

Assuming the input signal is modulated by a sinusoidal waveform

(10) $$\frac{d\theta_{in}}{dt}=\Delta \omega +Msin\omega_{m}t$$

(11) $$\Delta \omega =\omega_{in}-\omega_{out}$$

where $\omega_{m},\omega_{in},\omega_{out}$ and Δω are phase modulation, input signal, output signal angular frequencies and frequency detuning, respectively. The natural angular frequency and the damping coefficient are defined as follows:

(12) $$\omega_{n}=\sqrt{K_{0}/\tau_{1}}=2\pi f_{n}$$

(13) $$\zeta =\left(1+K_{0}\tau_{2}\right)/2\sqrt{K_{0}\tau_{1}}$$

To simplify the equations, the following normalized parameters are introduced:

(14a) $$\beta =\frac{\omega_{n}}{K_{0}}=\frac{1}{\sqrt{K_{0}\tau_{1}}}\;\mathrm{normalized}\;\mathrm{natural}\;\mathrm{frequency}$$

(14b) $$\sigma =\frac{\Delta \omega }{\omega_{n}}\;\mathrm{normalized}\;\mathrm{frequency}\;\mathrm{detuning}$$

(14c) $$\Omega_{m}=\frac{\omega_{m}}{\omega_{n}}\;\mathrm{normalized}\;\mathrm{modulation}\;\mathrm{frequency}$$

(14d) $$m=\frac{M}{\omega_{n}}\;\mathrm{normalized}\;\mathrm{maximum}\;\mathrm{angular}\;\mathrm{frequency}\;\mathrm{deviation}$$

By changing the time t into $\tau =\omega_{n}t$ and replacing τ by t again, Equation (9) can be given as

(15) $$\frac{d^{2}\phi }{dt^{2}}+\beta \left[1+\frac{\left(2\zeta -\beta \right)h^{\prime }\left(\phi \right)}{\beta }\right]\frac{d\phi }{dt}+h\left(\phi \right)=\beta \sigma +\beta msin\Omega_{m}t+m\Omega_{m}cos\Omega_{m}t$$

where $2\zeta -\beta =K_{0}\tau_{2}/\sqrt{K_{0}\tau_{1}}\geq 0.$ For simplicity, the filter is assumed to be a lag filter $2\zeta -\beta =0,\left(\tau_{2}=0\right)$. By changing the time t into t=t′−θΩm where tan(θ)=Ωmβ, and replacing $t^{\prime }$ by t again, Equation (15) can be simplified as

(16) $$\frac{d^{2}\phi }{dt^{2}}+\beta \frac{d\phi }{dt}+h\left(\phi \right)=\beta \sigma +asin\Omega_{m}t$$

(17) $$a=m\sqrt{\beta^{2}+\Omega_{m}{}^{2}}$$

**Figure 2:**
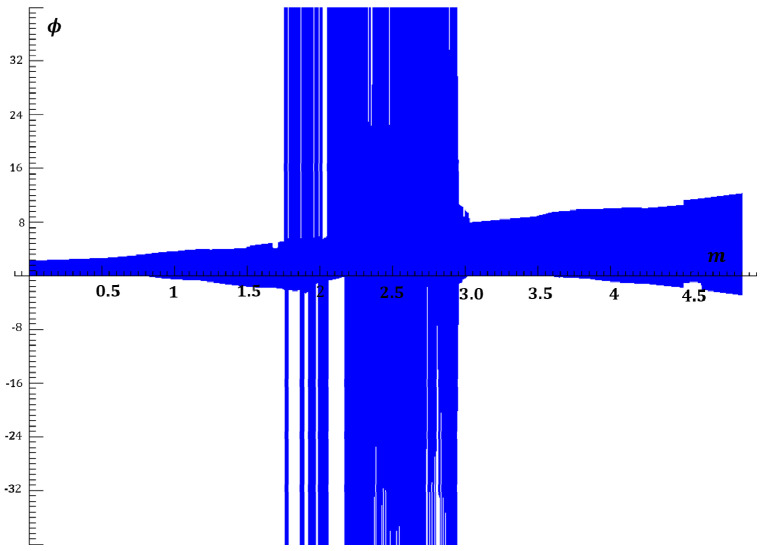
Bifurcation map of ϕ with respect to m illustrating bounded and unbounded chaos ($\beta =0.56,\;\;\Omega_{m}=0.9,\;\sigma =0$).

For small values of a, the solutions are periodic with $\Omega_{m}$, which means that the phase ϕ is phase-locked with the input signal. With an increase in a, the phase ϕ shows bifurcations and becomes chaotic, as seen in Figure 2. For fixed values of β and $\Omega_{m}$, the parameter m is gradually increased, thus the a parameter is linearly elevated. The bifurcations and transition from bounded to unbounded chaos is observed in Figure 2. It is observed that the PLL system demonstrates unbounded chaos when m (normalized maximum angular frequency deviation) is between approximately 1.75 and 3.

Depending on the parameters as seen in Figure 2, the chaos is either bounded or unbounded in the ϕ-direction. In [6,18,19,20], it is suggested that the chaotic change of *ϕ(t)* approaches a Wiener-Levy process over long times. Therefore, its derivative dϕ/dt is supposed to yield a white noise-like spectrum at angular frequencies substantially below $\Omega_{m},\;\;\omega_{o}\left(\omega_{o}=1\right)\;$and $\omega_{r}$($\omega_{r}=\beta /2$), where $\omega_{o}$ and $\omega_{r}$ are the natural angular frequency and the relaxation angular frequency of the simplified system, respectively. The chaotic signal at the VCO input is given as

(18) $${\dot{\theta }}_{out}=msin\Omega_{m}t-\dot{\phi }$$

In this paper, the focus is on the signal at the VCO input as it exhibits chaotic behaviors which can be exploited for random number generation. 

## 3. Chaotic Signal Formation

**Figure 3:**
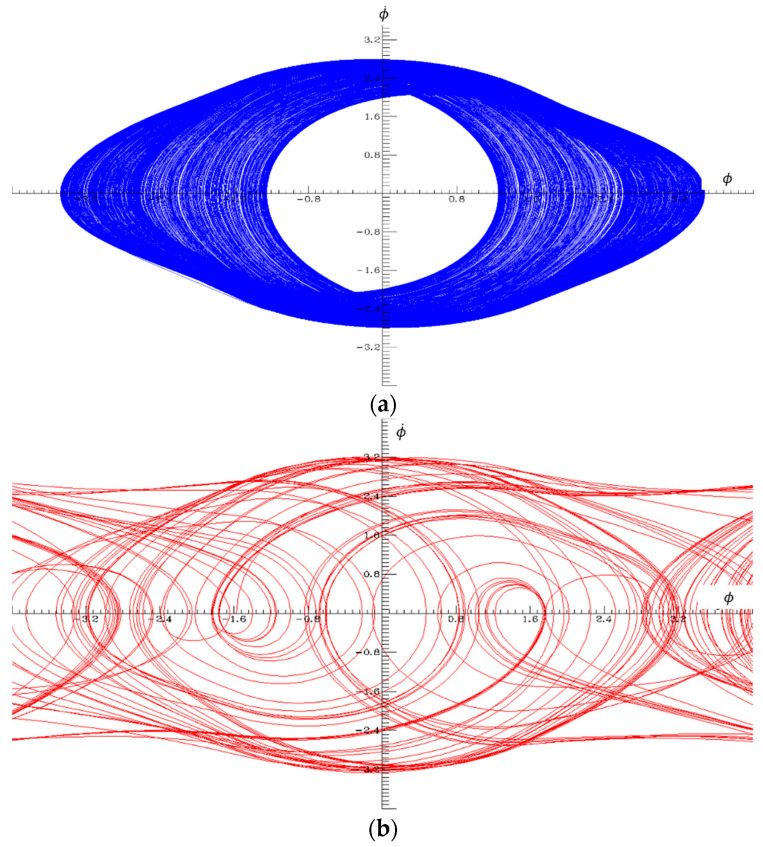
Lissajous patterns in $\phi -\dot{\phi }$ plane exhibiting (**a**) bounded and (**b**) unbounded chaos. Associated parameters $\beta =0.56,\;\Omega_{m}=0.9,\;\sigma =0$ and m=(1.74, 2.29) for bounded and unbounded chaos respectively.

In this section, the normalized equations of the PLL are entered into a numerical solver, and the system parameters are adjusted to set the PLL to operate in the chaotic regime. For numerical solutions, Dynamics Solver software is used. To put the system in chaos, the normalized natural frequency β and the normalized modulation frequency $\Omega_{m}$ are chosen to be 0.56 and 0.9, respectively, although many other combinations of parameter values for chaos can be found by experimenting with the numerical solver. Then, a is changed linearly by varying the m parameter according to (17). The bifurcation graph shown in Figure 2 suggests that for these values of β and $\Omega_{m}$, m=1.75 is the transition border from bounded to unbounded chaos. Figure 3 illustrates the change of Lissajous patterns in ϕ-$\dot{\phi }$ plane when the type of chaos transitions from bounded at m=1.74 to unbounded at m=2.29 by modifying only the *m* parameter in parallel with the bifurcation graph shown in Figure 2.

**Figure 4:**
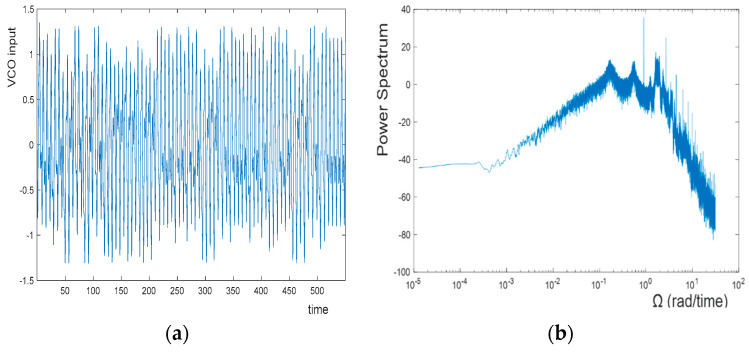
Chaotic signal at VCO input for bounded chaos (**a**) in the time domain, and (**b**) the associated power spectrum.

**Figure 5:**
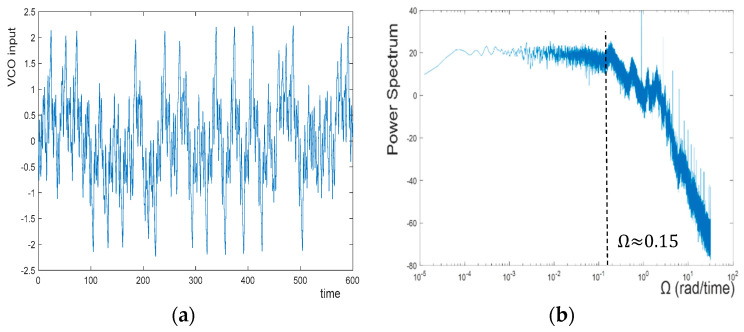
Chaotic signal at VCO input for unbounded chaos (**a**) in the time domain, and (**b**) the associated power spectrum.

Figure 4 and Figure 5 show the signals at VCO input in the time domain and the associated power spectra for cases of unbounded and bounded chaos in a comparative manner. As the solutions are obtained for normalized equations, units are not shown in Figure 4 and 5. Even by observing the time-domain waveforms, it may be possible to distinguish between unbounded and bounded chaos, as unbounded chaos displays a larger degree of irregularity and aperiodicity compared to bounded chaos. The time-domain signal at the VCO input, as shown in Figure 4, seems visually more regular compared to the signal shown in Figure 5. However, power spectral analysis is a safer method to identify the type of chaos. Figure 4 demonstrates that the power spectrum is not flat for bounded chaos; therefore, it is not an optimal entropy source for random number generation. However, for unbounded chaos, Figure 5 shows that the power spectrum for the VCO input in a normalized equation can be visually considered to be flat from DC up to approximately Ω≈0.15, which corresponds to a frequency of $f\approx 0.15\times f_{n}\approx 230\;\mathrm{Hz}$. To expand the flat spectrum range, it is necessary to increase the natural frequency for a fixed $\Omega_{m}$. Having a flat spectrum similar to white noise makes the VCO input signal from an unbounded chaos case a possible entropy source for the generation of random numbers using the regular sampling of an irregular waveform method. It exhibits very irregular and aperiodic behaviors, which make it unpredictable and useful to be exploited for random number generation.

## 4. Random Bit Generation and Discussion

A method to generate output bit sequence is regular sampling of the chaotic signal at the VCO input and comparing the samples with a threshold value. The ergodicity of chaotic signals makes it possible to analyze the distribution and statistical properties of the chaotic variable independent of initial conditions and sampling frequency. Therefore, the mean value of the samples over a long operation time can be selected as the threshold. At the time of sampling, if the signal value is below the threshold, bit *0* is produced; otherwise, the output is bit *1*. However, the sampling frequency $f_{s}$ needs to be adjusted to generate random bits with high entropy and without correlation between successive bits. The sampling frequency determines the throughput of the RNG, and it heavily depends on the power spectrum of the chaotic signal at the VCO input. For selection of the sampling frequency, autocorrelation analysis can be utilized as a metric to quantify randomness in a finite bit sequence. The absolute value of normalized autocorrelation function at a lag of one sampling period gives the correlation between successive bits. Therefore, bit sequences with a length of 20 KBits are generated by regularly sampling the chaotic signal at varying frequencies and the absolute value of the normalized autocorrelation at one-bit lag, i.e., one sampling period is calculated. To find the absolute value of the normalized autocorrelation at one-bit lag, first the mean of the 20 KBits sequence is subtracted from each binary value in the bit stream and $x_{n}$ is obtained. Then the autocorrelation function for the resulting bit sequence $x_{n}$ is normalized such that at zero lag, the value is 1, and then its absolute value is calculated. As the autocorrelation function for the 20 KBits sequence is real and symmetric around zero lag, the value corresponding to one-bit lag can be found by substituting m=±1 and taking the absolute value of the result in (20):

(19) $${\hat{R}}_{xx}\left(0\right)=\overset{N-m-1}{\underset{n=0}{\sum }}x_{n}{}^{2}\;$$

(20) $${\hat{R}}_{xx,normalized}\left(m\right)=\frac{1}{{\hat{R}}_{xx}\left(0\right)}\{\begin{array}{c} \overset{N-m-1}{\underset{n=0}{\sum }}x_{n+m}\;x_{n}\;\;,\;m\geq 0\\ {\hat{R}}_{xx}\left(-m\right)\;\;\;\;\;\;\;\;,\;m<0 \end{array}$$

The VCO input signals from the previous section depicting bounded and unbounded chaos are used for random bit generation by the regular sampling of an irregular waveform method. White Gaussian noise generated by Matlab is also used to generate random bits by regular sampling as a benchmark to assess the use of chaos as an entropy source for RNGs.

Figure 5 shows the relation between the sampling period and the absolute values of the normalized autocorrelation function at one-bit lag according to (20) for the bit sequences generated using bounded chaos, unbounded chaos and Gaussian white noise, respectively. As can be expected, the correlation between successive bits generally decreases when the sampling period increases, which also means reducing the sampling frequency. However, the plot of absolute values of the normalized autocorrelation exhibits local maxima and minima for both bounded and unbounded chaos. It is noteworthy to mention that the variation in the autocorrelation value is higher in the case of bounded chaos than for unbounded chaos. In case of bounded chaos, the peaks are separated from each other by approximately $3.6T_{n}$, where Tn=2πωn. Therefore, this can be interpreted as an indication that it would be easier to make a random generator by sampling the chaotic signal in unbounded chaos. For unbounded chaos, from Figure 5, it can be stated approximately that for obtaining a random bit stream, the sampling period should be adjusted as $T_{s}=k\cdot 5.\;2\cdot T_{n\;},k=2,\;3,\ldots$. Furthermore, the performance of unbounded chaos as an entropy source approaches that of white Gaussian noise. As white Gaussian noise is assumed to be white with infinite bandwidth, there is no restriction on the minimum sampling period that can be used to generate a random bit sequence. By proper selection of the sampling frequency of the chaotic signal, the absolute value of normalized autocorrelation values at one-bit lag of the bit sequence can be made close to that of the bit sequence generated by regular sampling of Matlab-based white Gaussian noise which has an infinite and flat power spectrum.

**Figure 6:**
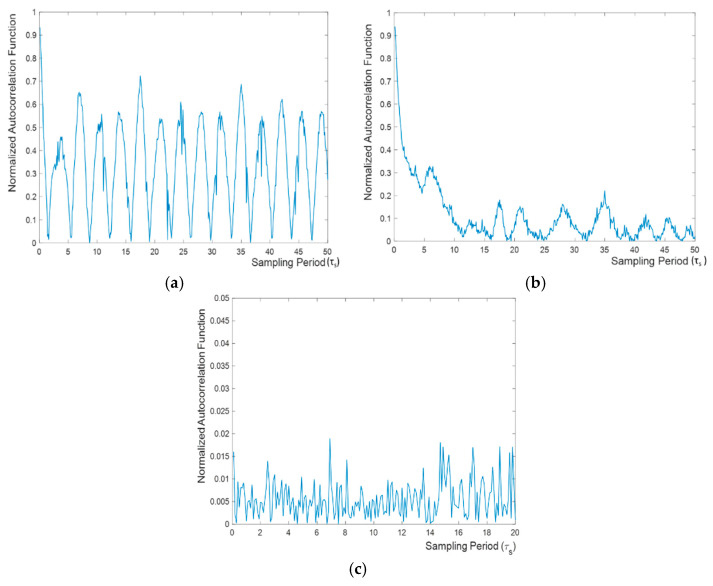
Relation between absolute value of normalized autocorrelation at one-bit lag and the sampling period *τ_s_* for (**a**) bounded chaos, (**b**) unbounded chaos, and (**c**) white Gaussian noise.

However, as can be observed from Figure 6, in general, the absolute value of autocorrelation of bit sequences at one-bit lag generated by white Gaussian noise are lower compared to bit sequences obtained from unbounded chaos. This is because the sampled chaotic signal is obtained purely by the solution of deterministic equations. However, if the system were implemented experimentally, the non-deterministic thermal and shot noise in electronic components would affect the chaotic trajectories continuously, making the bit stream generated by regular sampling of the chaotic signal non-deterministic, thus resulting in autocorrelation values closer to those of white Gaussian noise [9].

**Figure 7:**
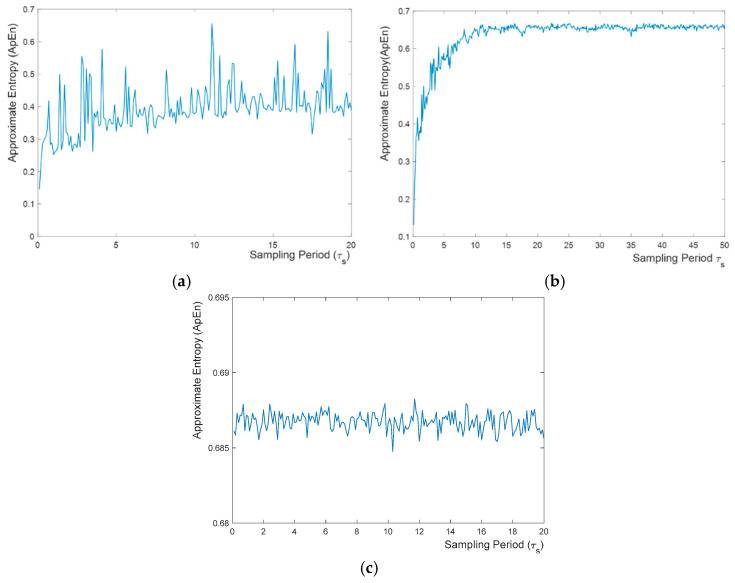
Relation between absolute value of normalized autocorrelation at one-bit lag and the sampling period *τ_s_* for (**a**) bounded chaos, (**b**) unbounded chaos, and (**c**) white Gaussian noise.

To further analyze the randomness of the RNG output, the concept of approximate entropy (ApEn) is used as a measure of sequential irregularity. For a finite length of bit sets, approximate entropy gives an idea of the randomness, with higher ApEn values indicating a higher level of randomness. ApEn approaches the theoretical maximum information entropy of ln(2)≈0.69 for a perfectly random bit sequence. The approximate entropy (ApEn) values of order 8 are calculated for the bit sequences of a length of 20 KBits generated by sampling unbounded chaos, bounded chaos and a Gaussian white noise signal at varying sampling frequencies. Figure 7 shows the relation between approximate entropy of the output bit stream and the sampling period for unbounded chaos, bounded chaos and Gaussian white noise, respectively. It is noteworthy to mention that at peak points of high autocorrelation, the approximate entropy is at a local minimum. Furthermore, increasing the sampling period, i.e., slowing the RNG throughput, generally results in higher ApEn value. In the case of bounded chaos, there exists a periodic pattern of ups and downs which is due to the non-flat power spectrum. However, in case of unbounded chaos, the ApEn value reaches the theoretical maximum quickly after a certain sampling rate, does not deviate very much, and approximates the value of white Gaussian noise. Unbounded chaos approaches white Gaussian noise, which shows the advantage of using unbounded chaos as an entropy source instead of bounded chaos.

As the amount of data that can be numerically produced with regular sampling of a chaotic PLL signal is limited; the data sequence is not put through NIST-800-22 test suite, since it involves at least 40 bit sequences with a length of 1Mbit. Instead, bit streams of 20 KBits length are subjected to tests of FIPS-140-2 test suite without any post processing [7]. The numerical binary streams of length 20 KBits obtained by regular sampling of the chaotic signal from a PLL device passed all 4 statistical tests, as shown in Table 1 where *p*-Value (0≤p-Value≤1) is a real number estimating the probability that a perfect RNG would produce a less random sequence than the given sequence.

## 5. Conclusions

In this paper, the chaotic signal from a phase-locked loop device is studied in order to develop a comparison between deterministic chaotic and stochastic processes for generation of random numbers. By adjusting the system parameters, the chaos becomes either bounded or unbounded in the phase direction, and this changes the power spectral characteristics of the chaotic signal. Random binary sequences are generated by regular sampling of the chaotic PLL signals. Autocorrelation and approximate entropy analysis is used to quantify the relationship between the randomness of the generated bit streams and the associated sampling periods. The chaotic signals are benchmarked against white Gaussian noise, and it is shown that unbounded chaos approaches Gaussian white noise as an entropy source for random number generation. To the best of the authors’ knowledge, this is the first analytical study investigating using bounded and unbounded chaos from a PLL device as an entropy source for random number generation and the comparison of deterministic chaos to white noise from a stochastic process.

## Figures and Tables

**Table 1:** Results of the FIPS-140-2 test suite for RNG based on regular sampling of chaotic signal.

Statistical Tests	*p*-Value
Frequency	0.777297
Block Frequency	0.543739
Runs	0.041646
Longest Run	0.496469
